# An Analysis of Physiology: Being a Condensed View of Its Most Important Facts and Doctrines

**Published:** 1848-06

**Authors:** 


ART. VI.—*An Analysis of Physiology:* being a condensed view of its most important Facts and Doctrines. Designed especially for the use of students. By JOHN J. REESE, M.D., Lecturer on Materia Medica in the Medical Institute of Philadelphia; Fellow of the College of Physicians, &c. Philadelphia, J. G. Auner, 1847.

This book was prepared at the solicitation of the author's class, and in the small compass of three hundred and twelve pages, contains about all that is known in Physiology. It will be found a most excellent companion for those attending lectures on that branch of medicine. C.

